# Variants of the *Matrix Metalloproteinase-2 *but not the *Matrix Metalloproteinase-9 *genes significantly influence functional outcome after stroke

**DOI:** 10.1186/1471-2350-11-40

**Published:** 2010-03-11

**Authors:** Helena Manso, Tiago Krug, João Sobral, Isabel Albergaria, Gisela Gaspar, José M Ferro, Sofia A Oliveira, Astrid M Vicente

**Affiliations:** 1 Instituto Gulbenkian de Ciência, Oeiras, Portugal; 2 Departamento Promoção da Saúde e Doenças Crónicas, Instituto Nacional de Saúde Dr Ricardo Jorge, Lisbon, Portugal; 3 Center for Biodiversity, Functional & Integrative Genomics (BIOFIG), Lisbon, Portugal; 4 Clinical Neurology Research Unit, Instituto de Medicina Molecular, Faculdade de Medicina da Universidade de Lisboa, Lisbon, Portugal; 5 Serviço de Neurologia, Hospital de Santa Maria, Lisbon, Portugal

## Abstract

**Background:**

Multiple lines of evidence suggest that genetic factors contribute to stroke recovery. The matrix metalloproteinases -2 (MMP-2) and -9 (MMP-9) are modulators of extracellular matrix components, with important regulatory functions in the Central Nervous System (CNS). Shortly after stroke, MMP-2 and MMP-9 have mainly damaging effects for brain tissue. However, MMPs also have a beneficial activity in angiogenesis and neurovascular remodelling during the delayed neuroinflammatory response phase, thus possibly contributing to stroke functional recovery.

**Methods:**

In the present study, the role of *MMP-2 *and *MMP-9 *genetic variants in stroke recovery was investigated in 546 stroke patients. Functional outcome was assessed three months after a stroke episode using the modified Rankin Scale (mRS), and patients were classified in two groups: good recovery (mRS ≤ 1) or poor recovery (mRS>1). Haplotype tagging single nucleotide polymorphisms (SNPs) in the *MMP-2 *(N = 21) and *MMP-9 *(N = 4) genes were genotyped and tested for association with stroke outcome, adjusting for significant non-genetic clinical variables.

**Results:**

Six SNPs in the *MMP-2 *gene were significantly associated with stroke outcome (0.0018<*P *< 0.0415), two of which survived the Bonferroni correction for multiple testing. In the subset of ischemic stroke patients, association of five of these SNPs remained positive (0.0042<*P *< 0.0306). No significant associations were found for the *MMP-9 *gene.

**Conclusions:**

The results presented strongly indicate that *MMP-2 *genetic variants are an important mediator of functional outcome after stroke.

## Background

While remaining one of the most common causes of death worldwide, stroke is also a leading cause of significant disability: after a first stroke event, 50-70% of stroke patients regain functional independence, but 15-30% are permanently disabled and 20% require institutional care at 3 months after onset [1]. Clinical and demographic factors can influence stroke outcome. In addition, genetic factors are likely to have an impact in stroke recovery processes and outcome: family history of stroke is associated with stroke outcome [2,3] and many animal models of stroke implicate genes that regulate angiogenesis, neuronal regeneration and proliferation, and neuroinflammation, in stroke recovery [4-7].

Several lines of evidence suggest that matrix metalloproteinases (MMPs) are fundamental players in stroke recovery. These molecules belong to a family of zinc-dependent endopeptidases that modulate extracellular matrix (ECM) components in many Central Nervous System (CNS) developmental and regenerative processes such as neurogenesis, axonal growth and regeneration, and myelin formation. The expression and activity of MMPs is tightly regulated. Most MMPs require proteolytic processing by proteases or other MMPs to become activated, and can be inhibited by tissue inhibitors of metalloproteinases (TIMPs). Dysregulated MMP activity will lead to uncontrolled degradation of ECM and basal lamina proteins, with serious harmful effects for the blood-brain barrier (BBB) integrity and neuroinflammatory or neurotoxic consequences [8,9]. Such dysregulation of MMPs is known to occur after stroke, leading to a degradation of the neurovascular matrix, disrupting cell-matrix homeostasis and weakening the BBB, and thus contributing to cell death, neurotoxicity, edema and hemorrhage [9,10]. The variation profiles of MMPs in blood after a stroke event [11,12] suggest that these molecules can eventually be used as biomarkers for brain damage and neurological outcome, while their contribution to tissue destruction renders MMPs inhibitors potentially interesting therapeutic targets for stroke.

Emerging studies, however, indicate that MMPs may also have a beneficial activity in angiogenesis and neurovascular remodelling during the delayed neuroinflammatory response phase after stroke, possibly contributing to stroke functional recovery [9]. While inhibition of MMP activity has consistently been demonstrated to be effective in reducing edema, infarct size and hemorrhagic transformation, some studies suggest the existence of a time window for these beneficial effects to take place [13,14].

In the present study we tested the impact of genetic variants in *MMP-2 *and *MMP-9 *in stroke recovery, in a population sample of 546 patients evaluated for stroke outcome at three months after the stroke event.

## Methods

Participants in the present study were recruited in the context of a wider research project to evaluate stroke risk factors in a Portuguese population sample, which enrolled first-ever stroke patients under 65 years of age through Neurology and Internal Medicine Departments of several hospitals in Portugal. Stroke was defined as a focal neurological deficit of sudden or rapid onset lasting more than 24 hours, and classified into ischemic or intracerebral hemorrhage based on brain imaging (computed tomography and/or magnetic resonance imaging). The diagnosis of stroke was confirmed by a neurologist. Demographic characteristics (age and gender), information on previous vascular risk factors and comorbid conditions (diabetes mellitus, hypertension, cardiac disease, dyslipidemia, obesity), life-style risk factors (smoking status, alcohol consumption, physical inactivity and others), and detailed clinical data during hospitalization, including neurological symptoms, complications and interventions, were collected for the majority of patients. Occurrence of aphasia, neglect, paresis, gaze paresis, dysphagia, permanent consciousness disturbance, urinary incontinence and medical and neurological complications were clinical parameters indicative of stroke severity. Stroke outcome at discharge and at three months was assessed, by direct interview, using the modified Rankin Scale (mRS).

For the present study, 568 patients with relevant clinical data and a DNA sample were available. Eight patients had a second stroke event after enrolment, affecting patient recovery, and were thus excluded. Of the remaining 560, 14 did not return after discharge for the three months evaluation, and therefore only 546 patients were included in the analysis. Patients were classified in two groups, according to their mRS at three months: patients with mRS ≤ 1 were assigned to the "good recovery" group and patients with mRS>1 were assigned to the "poor recovery" group (handicapped patients). 276 individuals were included in the good recovery group (63.0% males and 37.0% females) and 270 in the poor recovery group (64.4% males, 35.6% females). The poor recovery group included 12 patients who died before the three months evaluation (seven of them before hospital discharge, and five others after discharge). Genetic power calculations were performed using the CaTS software [15].

The study was approved by the Ethics Committee of Instituto Nacional de Saúde Dr. Ricardo Jorge and other hospitals involved, subjects gave informed consent and procedures followed were in accordance with institutional guidelines.

Single nucleotide polymorphisms (SNPs) within the *MMP-2 *and *MMP-9 *genes and up to 5 kb of the flanking regions were selected using the Haploview software (v4.0) [16], based on their tagging potential (HapMap Release 21/phase II July 2006). 4 SNPs in *MMP-9 *and 20 SNPs in *MMP-2 *were genotyped using the Sequenom iPLEX assays with allele detection by mass spectroscopy, using Sequenom MassARRAY technology (Sequenom, San Diego, USA) and following the manufacturer's protocol. Primer sequences were designed using Sequenom's MassARRAY Assay Design 3.0 software. 1 SNP in *MMP-2 *was genotyped using TaqMan^® ^Pre-Designed SNP Genotyping Assays, in an ABI PRISM 7900HT Sequence Detector System (Applied Biosystems, Foster City, USA). Extensive quality control was performed using eight HapMap individuals, duplicated samples within and across genotyping plates, Mendelian segregation in three pedigrees and no-template samples. Call rates <90% and deviation from Hardy-Weinberg equilibrium led to SNP exclusion from the analysis. 2 SNPs in *MMP-9 *failed quality control and were substituted. In total, 21 *MMP-2 *SNPs and 4 *MMP-9 *SNPs were analysed.

The effect of discrete and continuous non-genetic variables on stroke outcome at three months was determined using the Pearson χ^2 ^test and Mann-Whitney test, respectively. These included age, gender, stroke risk factors as well as data on clinical variables collected during hospitalization (like occurrence of paresis, aphasia and medical complications). Variables with a *P *< 0.25 in univariate analysis or of particular clinical relevance were included in a logistic regression model using forward selection [17] and were maintained in the model if they were associated at a *P *≤ 0.05 level with stroke outcome. Logistic regression analyses were then used to determine the effect of each genetic variable on stroke outcome after adjustment for those significant non-genetic variables. Odds ratio (OR) and 95% confidence intervals (95% CI) were computed for the log-additive model. Univariate and logistic regression analyses were performed using MASS and SNPassoc packages of the R software [18] (v2.6.0). The Gabriel et al. (2002) [19] default method of the Haploview software [16] (v4.0) was used to determine haplotype blocks in the *MMP-2 *and *MMP-9 *genes. Since recovery processes may be regulated differently in ischemic and hemorrhagic stroke patients, we performed the same analyses in the subset of ischemic stroke patients. The small number of hemorrhagic stroke patients (N = 105) precluded the independent analysis of this subset.

Significant associations in individual SNP analysis were corrected for multiple testing using the Bonferroni method. The alternative SNPSpD approach, based on the spectral decomposition (SpD) of matrices of pairwise linkage disequilibrium (LD) between SNPs was also applied [20]. Since some of the 21 SNPs genotyped in the *MMP-2 *gene are in LD with each other in our sample, we used the SNPSpD approach to estimate the effective number of independent SNPs in our sample for multiple testing corrections.

## Results

Clinical and demographic characteristics of the population sample are presented in Table 1. Univariate analysis showed that type of stroke and six clinical features indicative of stroke severity - occurrence of aphasia, urinary incontinence, paresis, consciousness disturbance, medical and neurological complications during hospitalization - were significant predictors of poor outcome. Sex ratio, age, and stroke risk factors were similar between the poor and good recovery groups, and approximately the same proportion of patients was being treated for hypertension in either group (34.0% and 34.6% in the good and poor recovery groups, respectively). Assuming an additive genetic model and disease allele frequency of 30%, our sample was 82% powered to detect a genotype relative risk of 1.5 with a type I error of 5%.

**Table 1:** Demographic and clinical characteristics of stroke patients.

Characteristic	Good Recovery (mRS ≤ 1)	Poor Recovery (mRS>1)	P*
**Age and Gender**			
Age, mean ± SD (yrs)	50.8 ± 9	52.5 ± 8.5	0.028
Gender (male), n/N (%)	174/276 (63.0)	174/270 (64.4)	0.734
			
**Past History, n/N (%)**			
Hypertension	159/241 (66.0)	143/240 (59.6)	0.147
Diabetes	36/259 (13.9)	47/246 (19.1)	0.115
Cardiac Disease	37/264 (14.0)	43/257 (16.7)	0.390
			
**Sroke type, n/N (%)**			
Ischemic stroke	238/276 (86.2)	193/270 (71.5)	-
Hemorrhagic stroke	33/276 (12.0)	72/270 (26.7)	-
Unknow type of stroke	5/276 (1.8)	5/270 (1.9)	-
			
**Stroke Features, n/N (%)**			
Aphasia	53/258 (20.5)	98/250 (39.2)	4.23 × 10^-6^
Neglect	11/266 (4.1)	19/240 (7.9)	0.072
Dysphagia	15/270 (5.6)	25/251 (10.0)	0.059
Urinary Incontinence	5/272 (1.8)	15/251 (6.0)	0.014
Paresis	203/273 (74.4)	244/269 (90.7)	5.59 × 10^-7^
Consciousness disturbance	21/275 (7.6)	59/265 (22.3)	1.72 × 10^-6^
Medical complications	18/265 (6.8)	82/254 (32.3)	1.83 × 10^-13^
Neurologic complications	14/274 (5.1)	39/267 (14.6)	2.03 × 10^-4^

*Mann-Whitney test or χ^2 ^test.

SD - standard deviation, yrs - years.

**Figure 1:**
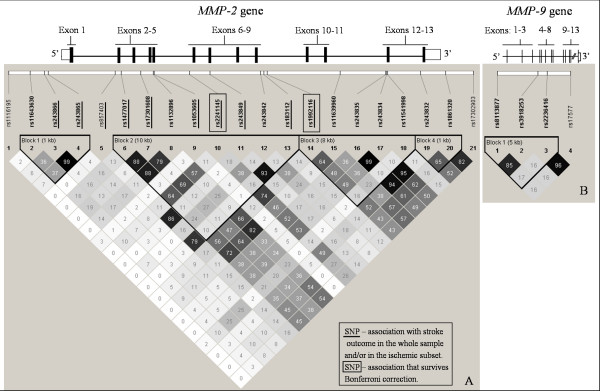
**Schematic diagrams of the *MMP-2 *(A) and *MMP-9 *(B) genes showing the location of the 13 exons (black boxes), the 5' and 3' untranslated regions (white boxes) and the pairwise r^2 ^plots for the 21 genotyped SNPs in *MMP-2 *and 4 genotyped SNPs in *MMP-9*, in our population sample**. Markers associated with three months outcome are indicated. Linkage disequilibrium blocks were generated using the Gabriel et al. [19] method.

Of 21 *MMP-2 *SNPs, six were associated with stroke outcome at three months under a log-additive model (0.0018<*P *< 0.0415) after adjusting for the significant covariates in a multivariate model: history of hypertension, type of stroke, occurrence of aphasia, paresis, consciousness disturbance and medical complications during hospitalization (Table 2; see Additional file 1). History of hypertension, although not associated in the univariate analysis, became significant in the multivariate model before inclusion of genetic variants, and was therefore included in the final regression model. SNPs rs2241145 and rs1992116 remained significantly associated with stroke outcome after Bonferroni correction for multiple testing (OR [95%CI] = 1.66 [1.20-2.30], corrected *P *= 0.0439, and OR [95%CI] = 1.67 [1.20-2.31], corrected *P *= 0.0385, respectively). Two haplotypes (one of which rare) were nominally associated with stroke outcome at three months (Table 3, Figure 1A; see Additional file 2).

**Table 2:** Genotype frequency distribution and association with stroke outcome at three months for *MMP-2 *SNPs.

		Whole sample*				Ischemic subset^†^			
			
		Genotype frequency				Genotype frequency			
							
SNP	Genotype	Good recovery, n (%)	Poor recovery, n (%)	OR [95%CI]	*P*	Good recovery, n (%)	Poor recovery, n (%)	OR [95%CI]	*P*
**rs243866**									
	G/G	142 (67.6)	117 (57.9)	1.67 [1.10-2.52]	**0.0143**	125 (67.6)	83 (56.8)	1.78 [1.13-2.80]	**0.0128**
	A/G	66 (31.4)	76 (37.6)			59 (31.9)	55 (37.7)		
	A/A	2 (1.0)	9 (4.5)			1 (0.5)	8 (5.5)		
									
**rs243865**									
	C/C	141 (67.8)	117 (57.9)	1.65 [1.09-2.50]	**0.0162**	124 (67.8)	83 (56.8)	1.76 [1.12-2.78]	**0.0143**
	C/T	65 (31.2)	76 (37.6)			58 (31.7)	55 (37.7)		
	T/T	2 (1.0)	9 (4.5)			1 (0.5)	8 (5.5)		
									
**rs857403**									
	A/A	124 (59.3)	138 (68.3)	0.71 [0.48-1.06]	0.0909	105 (57.1)	103 (70.5)	0.62 [0.40-0.97]	**0.0349**
	T/A	75 (35.9)	56 (27.7)			70 (38)	37 (25.3)		
	T/T	10 (4.8)	8 (4.0)			9 (4.9)	6 (4.1)		
									
**rs1477017**									
	A/A	100 (47.6)	81 (40.1)	1.42 [1.01-2.00]	**0.0415**	86 (46.5)	55 (37.7)	1.51 [1.04-2.20]	**0.0306**
	G/A	91 (43.3)	98 (48.5)			82 (44.3)	72 (49.3)		
	G/G	19 (9.0)	23 (11.4)			17 (9.2)	19 (13)		
									
**rs17301608**									
	C/C	94 (45.0)	75 (37.3)	1.40 [1.00-1.95]	0.0510	80 (43.5)	50 (34.5)	1.47 [1.01-2.12]	**0.0412**
	C/T	94 (45.0)	100 (49.8)			85 (46.2)	73 (50.3)		
	T/T	21 (10.0)	26 (12.9)			19 (10.3)	22 (15.2)		
									
**rs1053605**									
	C/C	188 (89.5)	170 (84.2)	2.02 [1.09-3.75]	**0.0227**	166 (89.7)	127 (87.0)	1.82 [0.93-3.58]	0.0817
	C/T	22 (10.5)	28 (13.9)			19 (10.3)	16 (11.0)		
	T/T	0 (0.0)	4 (2.0)			0 (0.0)	3 (2.0)		
									
**rs2241145**									
	G/G	79 (37.8)	56 (27.9)	1.66 [1.20-2.30]	**0.0021^‡^**	68 (37.0)	39 (26.9)	1.67 [1.17-2.40]	**0.0044**
	G/C	100 (47.8)	101 (50.2)			88 (47.8)	72 (49.7)		
	C/C	30 (14.4)	44 (21.9)			28 (15.2)	34 (23.4)		
									
**rs243849**									
	C/C	131 (62.7)	143 (71.5)	0.70 [0.46-1.07]	0.0948	112 (60.9)	108 (75)	0.59 [0.36-0.96]	**0.0314**
	T/C	70 (33.5)	52 (26.0)			65 (35.3)	33 (22.9)		
	T/T	8 (3.8)	5 (2.5)			7 (3.8)	3 (2.1)		
									
**rs183112**									
	G/G	134 (64.1)	145 (72.9)	0.66 [0.43-1.03]	0.0669	115 (62.5)	110 (76.9)	0.54 [0.32-0.90]	**0.0162**
	A/G	70 (33.5)	51 (25.6)			65 (35.3)	32 (22.4)		
	A/A	5 (2.4)	3 (1.5)			4 (2.2)	1 (0.7)		
									
**rs1992116**									
	G/G	87 (41.6)	65 (32.3)	1.67 [1.20-2.31]	**0.0018^‡^**	76 (41.3)	48 (33.1)	1.68 [1.17-2.42]	**0.0042**
	A/G	97 (46.4)	94 (46.8)			86 (46.7)	67 (46.2)		
	A/A	25 (12.0)	42 (20.9)			22 (12.0)	30 (20.7)		

95%CI - 95% Confidence Interval.

*OR [95%CI] and *P *for the log-additive genetic model after adjustment for significant covariates (history of hypertension, type of stroke, and occurrence of aphasia, paresis, consciousness disturbance and complications during hospitalization).

^†^OR [95%CI] and *P *for the log-additive genetic model after adjustment for significant covariates (history of hypertension, and occurrence of aphasia, paresis, consciousness disturbance and complications during hospitalization).

^‡^Significant result after Bonferroni correction.

Results were adjusted for significant covariates; Odds Ratio (OR)>1 indicates increased probability of poor recovery for the carriers of the minor allele; only associated SNPs are shown.

**Table 3:** Haplotype frequency distribution of the *MMP-2 *and -*9 *genes, and association with stroke outcome.

		Whole sample					Ischemic subset				
			
Gene	Haplotype	Haplotype frequency	Good recovery (%)	Poor recovery (%)	χ^2^	*P*	Haplotype frequency	Good recovery (%)	Poor recovery (%)	χ^2^	*P*
***MMP-2***	rs11643630-rs243866-rs243865	0.200	17.2	22.7	5.150	**0.0233**	0.198	16.8	23.5	6.125	**0.0133**
	TAT										
***MMP-2***	rs1477017-rs17301608-rs1132896-rs1053605-rs2241145-rs243849-rs243842-rs183112	0.011	1.8	0.4	4.776	**0.0289**	0.014	2.1	0.6	3.372	0.0663
	ACGCGTTG										
***MMP-9***	rs8113877-rs3918253-rs2236416	0.034	4.8	1.9	7.403	**0.0065**	0.038	4.9	2.4	3.680	0.0551
	TCA										

Only haplotypes with significant association results are presented.

The hypothesis that the recovery processes after ischemic and hemorrhagic stroke may be different and regulated by different sets of genes [21,22] led us to analyze the ischemic stroke subset independently. The haemorrhagic subset was too small for independent analysis (N = 105). In the ischemic stroke sample, five out of the previously associated SNPs in the *MMP-2 *gene remained significantly associated with stroke outcome at three months under a log-additive model (0.0042<*P *< 0.0306), after adjusting for the same significant covariates (excluding type of stroke) (Table 2; see Additional file 1). ORs for these SNPs in this subset were similar to the overall study sample. None of the SNPs remained significant after Bonferroni correction for multiple testing. However, when the SNPSpD method was used, taking into account regional LD patterns and therefore the number of SNPs which are effectively independent, the two SNPs that survived Bonferroni correction in the whole sample remained significant for the ischemic stroke subset (rs2241145 and rs1992116) (see Additional file 1). Four additional *MMP-2 *SNPs were nominally associated with ischemic stroke outcome at three months (0.0162<*P *< 0.0412, Table 2). Only one haplotype in *MMP-2 *was also associated (Table 3; see Additional file 2).

OR analysis indicates that, for the majority of significantly associated SNPs (including rs2241145 and rs1992116), carriers of the minor allele (less frequent allele) are significant predictors of poor outcome (OR>1); only for rs243842 in the whole population sample, and for rs857403 and rs183112 in the ischemic subset, carriers of the minor allele show an improved chance of good recovery from stroke (OR<1).

In the *MMP-9 *gene, one rare haplotype was associated with stroke outcome in the overall population sample (*P *= 0.0065, Table 3, Figure 1B; see Additional file 2), but no independent association was found for any of the four tested SNPs (see Additional file 1). No SNP or haplotype in the *MMP-9 *gene was associated with stroke outcome at three months in the ischemic subset (see Additional files 1 and 2).

None of the tested SNPs were associated with hypertension, indicating that the MMP-2 effect on recovery was not mediated by its role on vascular structure (data not shown).

Two of the *MMP-2 *SNPs (rs1053605 and rs243849) are located in exonic regions of the *MMP-2 *gene (exons 5 and 7, respectively), two SNPs (rs243866 and rs243865) are located upstream of the gene, and six SNPs are intronic (Figure 1A). Both nucleotide transitions in the exonic SNPs are silent. To investigate possible functional consequences for gene transcription of the two upstream SNPs (rs243866 and rs243865) and the two intronic SNPs that survived correction for multiple testing (rs2241145 and rs1992116), we conducted a bioinformatics search for putative transcription factor binding sites. The A allele of the upstream SNP rs243866 lies in the core of a sequence with high similarity to the matrix for two binding factors, the IPF1 (insulin promoter factor 1), and the POU5F1 (POU domain class 5 transcription factor 1). Both proteins are transcription activators. Since the AA and AG genotypes are more frequent in the poor recovery group, we can hypothesize that the presence of the A allele may lead to an increased transcription of the *MMP-2 *gene, and thus explain the negative impact on stroke recovery observed in this population sample. The presence of the T allele in the upstream rs243865 SNP forms a sequence with high similarity to the matrix for the PLZF binding factor (promyelocytic leukemia zinc finger protein), while the sequence containing the C allele has a stronger similarity with the matrix for the VDR/RXR (vitamin D hormone receptor/retinoid × receptor) heterodimer. However, both transcription factors act as repressors, and therefore these findings are more difficult to interpret. The rs2241145 and rs1992116 intronic SNPs did not contain sequences for any known putative transcription factor binding sites.

## Discussion

In the present study we show that *MMP-2 *gene variants are strongly associated with patient's functional disability at three months after stroke onset, in a large Portuguese population sample. Given the possible genetic heterogeneity in recovery processes after hemorrhagic and ischemic stroke [21,22], we also analysed the association of this gene with stroke outcome in the restricted subgroup of ischemic stroke patients. All but one *MMP-2 *gene variants associated with stroke in the overall population sample remained associated with ischemic stroke in this smaller subset. Additional markers were associated only in this subset, possibly reflecting the increased genetic homogeneity of the ischemic group in terms of recovery processes. Associated SNPs in the ischemic subset did not, however, withstand Bonferroni correction for multiple testing. This could reflect the reduction in power due to the smaller sample size in the restricted analysis and/or the overcorrection for the false positive rate that is the main frequent criticism for this method. In fact, the alternative SNPSpD approach [20], which takes into account LD patterns between genotyped SNPs in the tested population, may be more appropriate since the 21 genotyped *MMP-2 *SNPs are not independent; with this approach, the significance of association of two specific SNPs with stroke, in the ischemic subset or in the overall population sample, was retained after multiple testing correction. The association results after multiple testing correction, using the stringent Bonferroni method or the SNPSpD approach, strongly support a role for *MMP-2 *in stroke recovery. Validation through replication in a larger sample set by other groups is now advisable.

A limitation of the present study was the lack of availability of the National Institute of Health Stroke Scale (NIHSS) for these patients. To control for the effect of the severity of stroke in patients' outcome, we performed a logistic regression analysis using, as covariates, individual clinical variables associated with stroke clinical severity in our sample. Each selected variable was entered in the logistic regression model to identify those behaving as clinical predictors of stroke outcome. While this approach may not be as comprehensive as a widely used severity scale, it allowed us to include in the analysis parameters that reflect the severity of the event and, to a certain extent, patient's status at baseline.

While subject of controversy, the cut-off for the good and poor recovery groups was set between 1 and 2 because we chose to focus on a non-handicaped recovery group. According to Weisscher et al. (2008) [23], there is a clear lag on performance of outdoor activities between mRS 1 and 2, while between mRS 2 and 3 the major difference is the ability to perform complex activities of daily life, and thus a more clearly defined good outcome is given by setting the cut-off between mRS 1 and 2.

Multiple studies in animal models and humans have shown that the actions of MMPs contribute to BBB disruption and brain cell death, early after a stroke event. These damaging processes can be inhibited by MMP inhibitors, leading to reductions in infarct volume and significant improvements in behavioural scores compared with controls [10]. However, fitting with their role in development and regeneration, a beneficial influence of MMPs in the recovery processes that occur in later stages after a stroke event, including angiogenesis, remyelination, neural migration and general recovery of the neurovascular unit has been shown [13,14,24,25]. At present, we cannot dissect whether gene variation in *MMP-2 *is more important for the damaging effects in the earlier stages after stroke, or to the beneficial delayed responses, or both. Functional studies will be required to answer this question. However, the present findings may have important implications. On one hand it challenges the usefulness of MMP inhibitors for the treatment of stroke, not only because the time window of usefulness is likely limited, but also because it may depend on the individual's *MMP-2 *genotype. On the other hand, and given that MMP-2 has also been suggested to influence the risk of hemorrhagic transformation upon recombinant tissue plasminogen activator (tPA) therapy [26], it is a plausible hypothesis that treatment outcome may also be associated with *MMP-*2 gene variants. Further work needs to be carried out to elucidate these questions.

## Conclusions

The present study further reinforces the contribution of MMPs for stroke recovery by showing that specific *MMP-2*, but not *MMP-9*, gene variants influence stroke outcome. Replication of these associations in larger population samples, together with approaches that integrate evidence from multiple levels, including gene expression and functional analysis, will contribute for the validation of these results. Together with previous observations, the study leads to the hypothesis that individual variation in the *MMP-2 *gene may influence stroke treatment outcome.

## Competing interests

The authors declare that they have no competing interests.

## Authors' contributions

HM participated in the study design, carried out genotyping, performed the analysis and wrote the manuscript. TK carried out genotyping. JS carried out genotyping and performed the analysis. IA participated in the study design and sample collection. GG participated in the sample collection and databasing. JMF participated in the patient recruitment and evaluation, in the study design and revised the manuscript. SAO participated in the study design and revised the manuscript. AMV designed the study, performed the analysis, and wrote the manuscript. All authors read and approved the final manuscript.

## Pre-publication history

The pre-publication history for this paper can be accessed here:

http://www.biomedcentral.com/1471-2350/11/40/prepub

## Supplementary Material

Additional file 1 **Table 4: Association analysis results for *MMP-2 *and *MMP-9 *SNPs and stroke outcome**. Association analysis results for *MMP-2 *and *MMP-9 *SNPs and stroke outcome. Click here for file

Additional file 2 **Table 5: Association analysis results for *MMP-2 *and *MMP-9 *haplotypes and stroke outcome**. Association analysis results for *MMP-2 *and *MMP-9 *haplotypes and stroke outcome. Click here for file
